# The role of the pulmonary function laboratory in risk assessment for lung resection

**DOI:** 10.36416/1806-3756/e20250481

**Published:** 2026-03-05

**Authors:** Danilo C Berton, Denis E O’Donnell, José Alberto Neder

**Affiliations:** 1. Unidade de Fisiologia Pulmonar, Hospital de Clínicas de Porto Alegre, Universidade Federal do Rio Grande do Sul, Porto Alegre (RS) Brasil.; 2. Pulmonary Function Laboratory and Respiratory Investigation Unit, Division of Respirology, Kingston Health Science Center & Queen’s University, Kingston (ON) Canada.

## BACKGROUND

**Figure 1:**
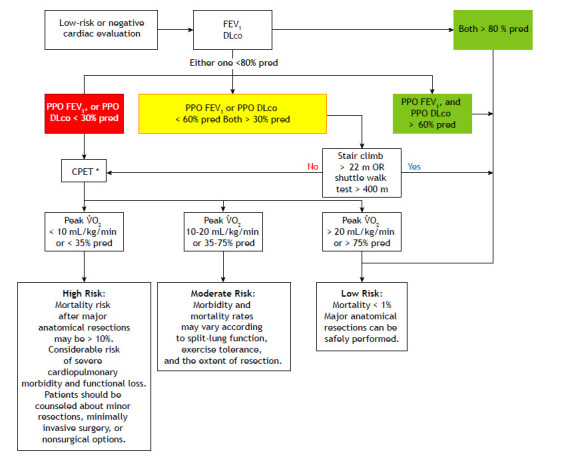
A simplified algorithm for physiological assessment before lung resection. Adapted from Brunelli et al.^1^ ^,^ ^2^ pred: predicted; PPO: percent predicted postoperative; and CPET: cardiopulmonary exercise testing.

Lung resection is often considered for patients with lung cancer and, in, some cases, for those with benign conditions such as localized bronchiectasis. Candidates for lung resection usually endorse respiratory symptoms and/or exhibit risk factors for impaired pulmonary function. Therefore, pulmonary function tests (PFTs) are essential tools in the preoperative assessment. The evaluation process includes assessing the likelihood of developing cardiopulmonary complications and the impact of the resection on postoperative lung function (Figure 1).^1^ ^,^ ^2^

*Clinical exercise tests peculiarities:

• Field tests are simpler and cheaper than CPET; however, they yield little physiological information regarding the specific mechanisms limiting exercise tolerance, which may influence risk prediction.

• CPET is especially useful for assessing patients at a high risk of cardiac events or with multiple comorbidities.

• Patients with a Minute Ventilation/Carbon Dioxide Production (V̇E/V̇CO_2_) slope > 35 L/L have been reported to have a significantly higher incidence of respiratory complications (22.0% vs. 7.6%) and mortality (7.2% vs. 0.6%) regardless of peak V̇O_2_ ^6^; however, V̇E/V̇CO_2_ might be blunted by significant mechanical ventilatory constraints in severe patients, underestimating the risks.^7^

• Oxygen desaturation > 4% from rest (but not isolated saturation < 90%) at maximal exercise is associated with postoperative cardiopulmonary complications. However, care should be taken because, in comparison with walking, cycling-based tests may underestimate the severity of hypoxemia.

• The risk of postoperative pulmonary complications may be higher in those with mechanical ventilatory constraints for a similar peak V̇O_2_; this highlights the importance of measuring inspiratory capacity during exercise.

• Given a high V̇O_2_ at a given work rate, reliance on peak V̇O_2_ may be misleading in obese patients (i.e., those with a BMI > 35 kg/m^2^) in the presence of increased work of breathing or mechanical inefficiency during cycling or walking. In such contexts, peak work rate should also be considered.

## OVERVIEW

A 75-year-old male with a 140 pack-year smoking history was referred for PFTs before surgery because of a 4 cm (stage IB) adenocarcinoma (cT2aN0M0) in the right upper lobe. He reported dyspnea with moderate exertion (a modified Medical Research Council dyspnea scale score of 2). There was no history of exacerbations on current inhaled triple therapy. Spirometry showed a moderate obstructive ventilatory defect (an FEV_1_ of 56% of the predicted value and an FEV_1_/FVC ratio of 0.40) with moderate decrements in DL_CO_ (= 48% of the predicted value). On the basis of these results, he was referred for a cardiopulmonary exercise test (CPET). Main results included peak V̇O_2_ of 1,329 mL/min (94% of the predicted value and 19.8 mL/kg/min), normal SpO_2_ (rest SpO_2_ = 95%; peak SpO_2_ = 94%), mildly increased exercise ventilation (lowest point of the ventilatory equivalent for carbon dioxide = 35 L/L), and, importantly, preserved mechanical inspiratory reserves at peak exercise (tidal volume/inspiratory capacity = 0.65). Right upper lobectomy was successfully performed without complications. 

Spirometry and DL_CO_ should be routinely measured in all patients undergoing lung resection.^1^ ^,^ ^2^ Lower FEV_1_ ^3^ and DL_CO_ ^4^ are associated with a higher risk of postoperative pulmonary complications (such as pneumonia, respiratory failure, and acute respiratory distress syndrome), which ultimately lead to higher health care costs and increased mortality risk.^5^ Patients with a preoperative FEV_1_ or DL_CO_ < 80% of the predicted value should undergo further testing. Percent predicted postoperative lung function accounts for preoperative values; the amount of lung tissue to be resected; and the contribution of the resected tissue to overall lung function. Lung segment counting by means of routine preoperative chest CT is more suitable for patients undergoing lobectomy or segmentectomy. This is due to challenges with scintigraphy, which is preferred for pneumonectomy, in assessing the contribution of individual lobes to overall perfusion. With the use of the first method, the estimated postoperative parameter is calculated as follows:

$$\;Percent\;predicted\;postoperative\;lung\;function\;=\;preoperative\;value\;\times \left(\frac{\;1\;-\;the\;number\;of\;functional\;segments\;to\;be\;re\sec ted}{\begin{array}{c} \;total\;number\;of\;unobstructed\;segments\\ \left(typically\;10\;on\;the\;right\;+\;9\;on\;the\;left\;=\;19\right) \end{array}}\right)$$

Based on indirect evidence and expert consensus,^1^ ^,^ ^2^ cutoff values for percent predicted postoperative lung function have been proposed to guide the use of clinical exercise tests in risk stratification (Figure 1). In the present case, the percent predicted postoperative FEV_1_ and DL_CO_ were 47% and 40%, respectively. Despite these concerning values, the CPET indicated a low risk. It is of note that only mild excess ventilation, normoxemia, and the absence of ventilatory constraints were reassuring relative to the gas exchange and mechanical reserves of the patient. 

## CLINICAL MESSAGE

Despite being recommended for all patients, PFTs are particularly relevant for those with preexisting lung conditions or respiratory symptoms that may impact the tolerability of lung resection. Clinical exercise tests are instrumental for patients with borderline resting PFTs (percent predicted postoperative lung function < 60%) and those with a high cardiac risk. A CPET offers the critical advantages of detecting clinically occult heart disease that may only manifest under stress; and helping differentiate the primary mechanisms and magnitude of exercise limitation. This detailed understanding is paramount for risk stratification and potential optimization of the preoperative condition of the patient. 
